# Effectiveness and Acceptability of a Mobile Phone Text Messaging Intervention to Improve Blood Pressure Control (TEXT4BP) among Patients with Hypertension in Nepal: A Feasibility Randomised Controlled Trial

**DOI:** 10.5334/gh.1103

**Published:** 2022-02-23

**Authors:** Buna Bhandari, Padmanesan Narasimhan, Rohan Jayasuriya, Abhinav Vaidya, Aletta E. Schutte

**Affiliations:** 1 School of Population Health, Faculty of Medicine and Health, University of New South Wales, Sydney, NSW, Australia; 2 Central Department of Public Health, Tribhuvan University Institute of Medicine, Kathmandu, Nepal; 3 Department of Community Medicine, Kathmandu Medical College and Teaching Hospital, Kathmandu, Nepal; 4 The George Institute for Global Health, Sydney, NSW, Australia

**Keywords:** Mobile health, SMS, Adherence, mHealth, Blood pressure

## Abstract

**Background:**

Uncontrolled blood pressure (BP) is the leading cause of preventable deaths in low- and middle-income countries. mHealth interventions, such as mobile phone text messaging, are a promising tool to improve BP control, but research on feasibility and effectiveness in resource-limited settings remains limited.

**Objective:**

This feasibility study assessed the effectiveness and acceptability of a mobile phone text messaging intervention (TEXT4BP) to improve BP control and treatment adherence among patients with hypertension in Nepal.

**Methods:**

The TEXT4BP study was a two-arm, parallel-group, unblinded, randomised controlled pilot trial that included 200 participants (1:1) (mean age: 50.5 years, 44.5% women) with hypertension at a tertiary referral hospital in Kathmandu, Nepal. Patients in the intervention arm (n = 100) received text messages three times per week for three months. The control arm (n = 100) received standard care. The COM-B model informed contextual co-designed text messages. Primary outcomes were change in BP and medication adherence at three months. Secondary outcomes included BP control, medication adherence self-efficacy and knowledge of hypertension. A nested qualitative study assessed the acceptability of the intervention.

**Results:**

At three months, the intervention group had greater reductions in systolic and diastolic BP vs usual care [–7.09/–5.86 (p ≤ 0.003) vs –0.77/–1.35 (p ≥ 0.28) mmHg] [adjusted difference: systolic β = –6.50 (95% CI, –12.6; –0.33) and diastolic BP β = –4.60 (95% CI, –8.16; –1.04)], coupled with a greater proportion achieving target BP (70% vs 48%, p = 0.006). The intervention arm showed an improvement in compliance to antihypertensive therapy (p < 0.001), medication adherence (p < 0.001), medication adherence self-efficacy (p = 0.023) and knowledge on hypertension and its treatment (p = 0.013). Participants expressed a high rate of acceptability and desire to continue the TEXT4BP intervention.

**Conclusion:**

The TEXT4BP study provides promising evidence that text messaging intervention is feasible, acceptable, and effective to improve BP control in low-resource settings.

**Trial registration:**

*anzctr.org.au* Identifier ACTRN12619001213134.

## Introduction

The burden of hypertension is escalating globally, especially in low- and middle-income countries (LMICs) in South-East Asia and Sub-Saharan Africa [1,2]. Uncontrolled blood pressure (BP) among patients diagnosed with hypertension is a significant challenge [3], leading to high rates of morbidity and mortality largely due to stroke and ischemic heart disease [4]. Nepal is a LMIC in South Asia with an estimated population of 29 million people. It had an overall pooled prevalence of hypertension of 28.5% in 2021 [5]. However, around half of patients in Nepal diagnosed with hypertension have uncontrolled BP [5,6], significantly contributing to mortality rates and the burden of the disease [7]. Major contributors to uncontrolled BP are poor adherence to antihypertensive medication [8], unhealthy diet (high salt, low fruit and vegetable intake) [9] and physical inactivity [10]. This has been confirmed by earlier work that we and others have done in Nepal [11,12].

The 2020 International Society of Hypertension Guidelines recommends a treatment target of <130/80 mmHg for patients younger than 65 years and strongly encourages all BP control efforts [13]. Patient-focused strategies for improving BP control require a systematic approach that should include adequate support for behaviour modification and improved knowledge [14]. In the context of low clinician-to-patient ratios to deliver intensive interventions in LMICs, novel alternatives need to be considered.

Although LMICs face many financial and health system challenges, the rapid uptake and availability of mobile phones provides excellent opportunities for a host of interventions as they are widely integrated into daily life, also in Nepal [15]. Mobile technologies have great potential to deliver healthcare messages effectively and offer a promising tool in resource-limited settings [16]. Previous studies have reported favourable outcomes in using text messages (SMS) in managing hypertension, namely promoting lifestyle change [17], improving medication adherence [18,19], and managing cardiovascular risk factors [20,21]. However, these studies are mostly confined to high-income countries that differ substantially from LMICs.

The potential of mobile phones in the management of hypertension has yet to be evaluated in Nepal. Our formative work in Nepal [22,23] found health workers and patients with hypertension to be highly receptive towards mobile phone interventions, such as messaging to reinforce the value of medication adherence (based on theories of behaviour modification). We therefore assessed the effectiveness and acceptability of a text message intervention to improve BP control (TEXT4BP) and treatment adherence among patients with hypertension attending a tertiary hospital in Nepal.

## Methods

### Study design and setting

This pilot study was conducted using a two-arm, parallel-group, unblinded, randomised, controlled trial among patients with diagnosed hypertension. We also used a nested qualitative design to assess the acceptability of the TEXT4BP intervention among the intervention arms. This study was conducted in the Cardiology and Medicine Outpatient Department (OPD) of the Kathmandu Medical College and Teaching Hospital (KMCTH), Nepal. At the KMCTH, an average of 15–20 adults with diverse backgrounds (all income groups) from various parts of Nepal seek service for the management of hypertension at the Cardiology/Medicine OPD per day. The methods and design have been described previously [24].

### Sample size

The sample size was based on recommendations for determining the sample size for a pilot randomised control trial [25]. For the main trial to have 90% power, two-sided alpha of 0.5 and standardised effect size to be small (0.1), a pilot trial with a sample size of 75 patients per treatment arm (1:1) was recommended. We estimated the attrition rate to be 30%, and therefore included 100 participants in each arm.

### Study population and recruitments

We included patients diagnosed with hypertension aged 18–69 years and prescribed for antihypertensive medication for at least three months. Eligibility criteria included that participants had a mobile phone and were able to read text messages by themselves or with their family’s help. We excluded patients with severe physical or mental illness, which would reduce their ability to participate in the study. Pregnant and post-partum women were also excluded from the study. All eligible patients were invited to the study by a relevant health professional (cardiologist, physician, nurse) who provided information about the study using a leaflet. Those who showed interest were asked to contact the research team directly for participation. Once the interested participants initiated contact, the researchers made an appointment, described the study, obtained written informed consent, and carried out a baseline data collection. Randomisation was done after baseline data collection.

### Randomisation and allocation

We used a simple randomisation technique [26] and allocated participants using an opaque sealed envelope [27] in the intervention or control arm. This sealed envelope method is one of the methods suggested by Schulz and Grimes [28] for the allocation concealment to prevent allocation bias.

### TEXT4BP intervention procedure (described in detail previously) [24]

Intervention development and delivery were informed by formative qualitative studies [22,23], and used a well-tested model of behaviour change, the COM-B model (Capability, Opportunity and Motivation- Behaviour) of the behaviour change wheel and behaviour change techniques (BCTs) [29]. We first mapped the enablers and barriers for medication adherence and change in lifestyle identified in the formative study [22] under the components of COM-B model. The content of the SMS intervention was developed using relevant behaviour change techniques (BCTs) to systematically address enablers and barriers. Some examples are presented in the supplementary file 1. The intervention arm received mobile phone text messages containing (a) general patient educational information (hypertension and its treatment, complications, signs and symptoms, medication, common side effects and consequences of non-adherence, physical activity, diet low in salt, low fat and some cultural messages) and (b) reminders for taking medication. Smoking- and alcohol-related messages (c) were tailored according to each patient. Text messages were sent three times per week in the morning (9–10 am) for three months, with the average length of the text message being 160 characters (see Supplementary file 1: Sample of the intervention text messages). We engaged a company (Aakash SMS: *https://aakashsms.com/*) to deliver the text messages which use the Nepal Telecom and N Cell telecom providers.

### ‘Usual care’

The control group received standard care. Patients with hypertension receives a prescription of medicine and advice for follow-up in Nepal. At the end of the study, we provided all control participants with a pamphlet containing educational information on hypertension and recommended modification of lifestyle and health behaviours.

### Data collection methods

Baseline (0 weeks) and follow-up (12 weeks) data collection were conducted using a tablet-based KOBO tool box (*https://www.kobotoolbox.org*), containing questions of sociodemographic characteristics, family and medical history, literacy status (literate: able to read and write, illiterate: not able to read and write) and lifestyle factors. These factors included: dietary salt consumption, physical activity, smoking status and alcohol intake based on the World Health Organisation STEPWISE (WHO STEPs) survey [30], the Hill Bone Compliance to High BP Therapy Scale [31], Medication adherence self-efficacy scale [32] and a researcher-developed structured questionnaire on the knowledge of hypertension and its treatment.

The Hill Bone compliance scale [31] is used to measure adherence to antihypertensive therapy. It consists of 14 items: nine items on medication, three items on salt intake and two items on appointment keeping. Each item is scored on a response of 1–4. The total score of Hill Bone compliance tool is 56, where total medication related score is 36, total salt intake score is 12 and appointment keeping score is 8, where a lower score indicates higher adherence to antihypertensive therapy. In the current study, the reliability coefficient Cronbach’s alpha of the total Hill Bone compliance scale was 0.87 at baseline and 0.92 at follow-up. The medication adherence self-efficacy tool had 13 items, where the score ranged from 1–4, with a higher score indicating better medication adherence self-efficacy. Cronbach’s alpha of the medication adherence self-efficacy tool’s reliability coefficient was 0.98 at baseline and 0.97 at the follow-up. During follow-up, participants in the text message intervention group were also asked about the acceptability of the intervention using the Marshfield usability survey tool [33]. The tool contains 13 items, and each item has a 1–4 response. We also conducted in-depth qualitative interviews among five intervention arm participants, selected purposively based on their age [18–45yrs (N-2), 46–60 yrs (N-2), 61–69yrs (N-1)], sex [male (N-2), female (N-3)], and literacy level [literate (N-2), illiterate (N-3)]. All the study tools were pretested, and necessary modification was done before the actual data collection.

### Physical measurements

BP, height, weight, waist and hip circumference were measured by a trained research assistant (clinically trained registered nurse) following a standard protocol. Brachial BP was measured with the participant in the sitting position using an oscillometric BP monitor (Kenz BPM OS-30, Suzuken Pvt. Ltd, Japan) following the guidelines of the European Society of Hypertension [34]. Patients were rested for 5 min, and three systolic and diastolic readings were measured 1–2 min apart, and the mean of the last two readings was used. A digital scale and height stadiometer were used for the weight and height measurement respectively. Waist and hip circumference were measured using a constant tension measuring tape using standard procedures.

### Outcomes

The primary outcomes of the study were change in the systolic and diastolic BP, and medication adherence score from baseline to follow-up. The secondary outcomes were changes in BP control, medication adherence self-efficacy score and knowledge score of hypertension. Acceptability of the intervention was also one of the secondary outcomes of the study.

### Statistical analyses

All analyses were done in SPSS version 26 (IBM Corp, NY/USA) using the intention to treat analysis approach. Baseline and follow-up sociodemographic information and outcomes were compared using the Chi-square test for categorical data and independent sample t-tests for continuous data. We analysed the three-month primary outcomes using a linear mixed model for repeated measures, which included data available on all randomised patients attending the follow-up visit at three months. This method has the advantage of implicitly accounting for missing data at the random mechanism using maximum likelihood. We also included an interaction term between the time and randomised group to assess possible differences of treatment effects (text message intervention vs usual care) per time points.

Additionally, we adjusted the model for other covariates, though there were no differences between the groups at baseline. As a measure of the intervention effect, the estimated mean difference (β) between groups alongside 95% confidence intervals (CIs) and two-sided p values were reported, and statistical significance was set at p < 0.05. Histograms and scatterplots of residuals and the predicted value suggested a good model fit. Further, a sensitivity analysis [35] was conducted using multiple imputations to create 20 imputed datasets of the missing values at follow-up. Univariate and multivariate general linear model analysis was performed to determine the robustness of the findings. In-group comparisons were conducted using the paired *t-test*. Qualitative interviews were transcribed, and line-by-line inductive coding was completed. Analysis was facilitated with NVivo software V.12 (QSR International, London, UK and USA).

### Patient and public involvement

Formative studies [22,23] informed our intervention through input from patients with hypertension, health care providers and key informants. However, participants were not invited to comment on the study design or contribute to research results and dissemination.

### Ethics and dissemination

This study was approved by the University of New South Wales Human Research Ethics Committee B (HC190357), Nepal Health Research Council (302/2019) and Institutional Review Committee of Kathmandu Medical College and Teaching Hospital Kathmandu, Nepal (030520192). All participants provided written informed consent.

## Results

**Figure 1:**
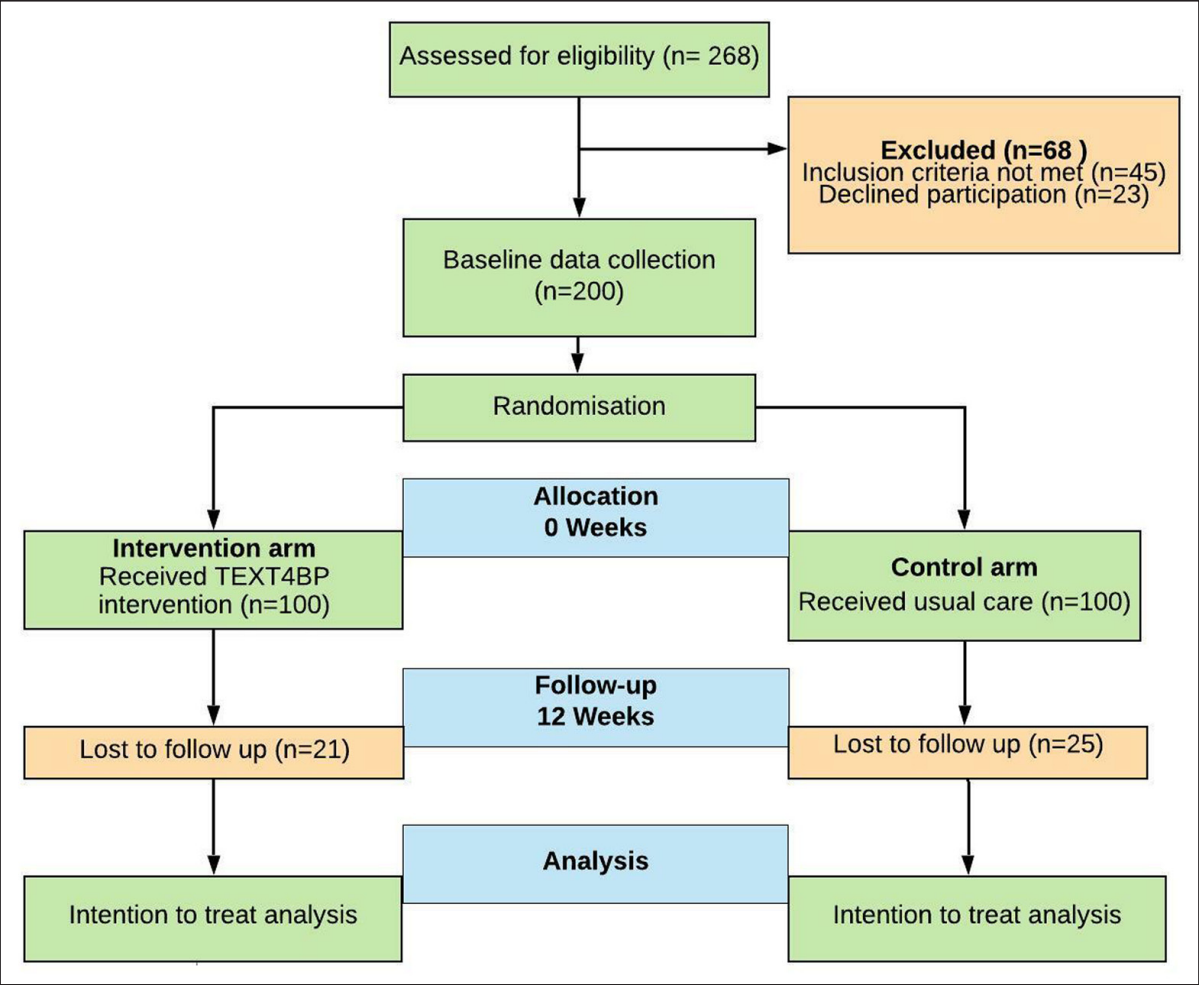
CONSORT flow diagram of the TEXT4BP Study.

A total of 268 patients with hypertension were screened and assessed for eligibility (see CONSORT flow diagram in ***Figure 1***). Of these, 45 did not meet the inclusion criteria, and 23 [No time for data collection (N = 14), not interested (N = 9)] declined to participate. Thus 200 participants were included in the TEXT4BP trial after study procedures were explained in detail and written informed consent was obtained. None of the recruited participants refused to receive the intervention or withdrew their consent during the study. Over the 12 weeks of follow-up, the retention rate was 77%. The reasons for loss to follow up were; mobile number unreachable (N = 8), could not come for follow up or busy (N = 11), restricted contact due to COVID-19 lockdown (N = 26). We compared the sociodemographic variables between those completing the study and those lost to follow-up and found no differences (all p ≥ 0.15, see Supplementary file 2: STable 1). A total of 18% (n = 18) of the intervention arm participants received the intervention with family support.

### Baseline characteristics of the study participants

The intervention and control groups had a similar distribution in terms of age (with more than 50% of the participants between 46–60 years), sex, literacy, marital status, and religion (***Table 1***). All socioeconomic, blood pressure, and behavioural characteristics were comparable (p ≥ 0.20) between the intervention and control arm, except for the Hill Bone salt-related score, which was slightly higher in the intervention arm (p = 0.043).

**Table 1:** Comparison of baseline sociodemographic and outcome measures in the intervention and control arm.


CHARACTERISTICS	CATEGORY	INTERVENTION ARM N = 100	CONTROL ARM N = 100	P VALUE

**Age (years)**	18–45	32	23	0.28a

	46–60	57	61	

	61– 69	11	16	

	Mean ± SD	49.2 ± 9.78	51.7 ± 9.21	0.071

**Sex, n**	Female	42	47	0.48 a

**Literacy status,+ n**	Literate	82	72	0.091 a

Ethnicity*, n	Upper caste groups	63	60	0.34 a

	Relative advantages and disadvantages Janajatis	29	36	

	Others**	8	4	

**Marital status, n**	Married	88	92	0.35 a

**Employment, n**	Employed	53	55	0.77 a

**Religion, n**	Hindu	92	91	0.80 a

	Others	8	9	

**Systolic BP (mmHg)**	Mean ± SD	134 ± 19.5	137 ± 25.3	0.38 b

**Diastolic BP (mmHg)**	Mean ± SD	84 ± 11.6	86 ± 13.4	0.35 b

**BP control (<140/90 mmHg)**	N (%)	60	54	0.39 a

**Duration of hypertension (years)**	Mean ± SD	5.40 ± 6.02	6.50 ± 6.18	0.20a

**Antihypertensive agents used**	One	73	74	0.87 a

	More than one	27	26	

Other comorbidities***	Yes	56	62	0.39 a

**Hill Bone Compliance score**				

**Total score (56)**	Mean ± SD	25.9 ± 6.10	25.5 ± 6.13	0.61 b

– **Medication related score (36)**	Mean ± SD	14.6 ± 4.77	14.0 ± 5.07	0.43 b

– **Salt related score (12)**	Mean ± SD	7.06 ± 1.36	7.02 ± 1.31	0.043 b

– **Appointment related score (8)**	Mean ± SD	4.35 ± 0.98	4.50 ± 0.97	0.28 b

**Medication adherence self-efficacy score**	Mean ± SD	36.6 ± 7.87	36.6 ± 9.68	0.95 b

**Knowledge of hypertension score**	Mean ± SD	17.5 ± 3.75	17.3 ± 3.92	0.66 b


*Note*: Number and percentage are the same as the denominator is 100 for each. ^a^ p value of Chi-square test, ^b^ p-value of the t-test * caste classification card used in STEPS survey Nepal is used for ethnicity division with six caste category [54]. ** others = (Dalit, disadvantaged non-Dalit Terai caste groups, religious minorities) *** other comorbidities: Diabetes, COPD, Arthritis, etc., + Literate: Ability to read and write.

### Effects of the intervention

#### Blood pressure

**Figure 2:**
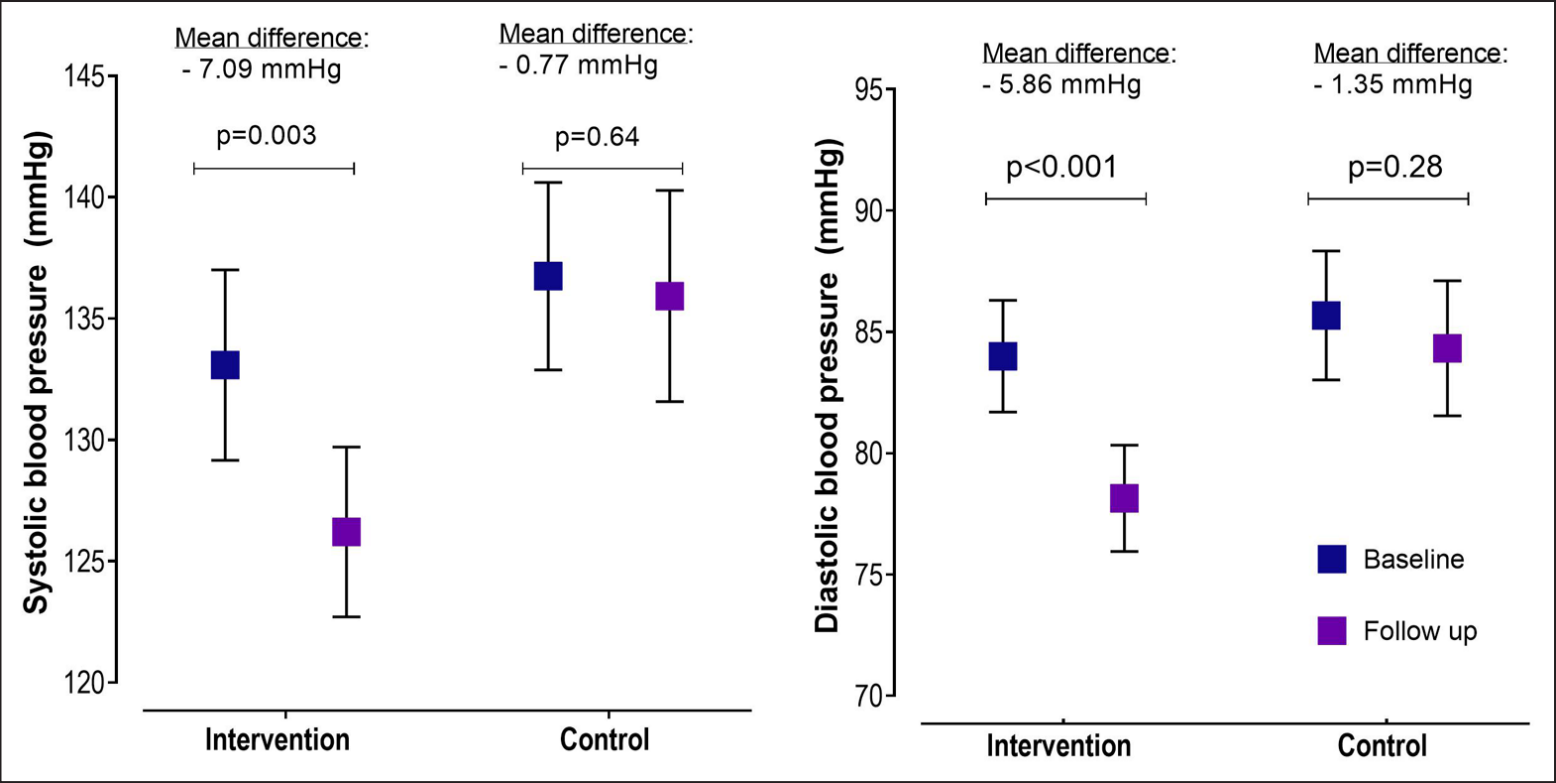
Change in systolic and diastolic blood pressure from baseline to follow-up in the text message intervention and control groups.

We found a significant decrease in systolic (p = 0.003) and diastolic BP (p < 0.001) in the intervention arm only, at follow-up compared to baseline (***Figure 2***).

At follow-up, systolic (p = 0.001) and diastolic BP (p < 0.001) were significantly lower in the intervention arm when compared to the control arm (***Table 2***). The intervention arm had a greater reduction in systolic (β = –6.36, p = 0.043) and diastolic (β = –4.51, p = 0.013) BP at follow-up than those in the control arm. These BP reductions remained significant after adjusting for age, sex, education, marital status, occupation, religion, smoking status and alcohol intake (***Table 2***).

**Table 2:** Effectiveness of the TEXT4BP intervention among the intervention group compared to the control group.


OUTCOME VARIABLE	INTERVENTION (12 WEEKS) M (SD)	CONTROL (12 WEEKS) M (SD)	P VALUE^a^	TIME*GROUP^b^ REGRESSION COEFFICIENT MODEL 1			TIME*GROUP^b^ REGRESSION COEFFICIENT MODEL 2	
	
				*B (95% CI)*	*P VALUE*		*B (95% CI)*	*P VALUE*

Systolic BP	126 ± 15.6	136 ± 18.9	0.001	–6.36 (–12.5, –0.19)	0.043		–6.50 (–12.6, –0.33)	0.039

Diastolic BP	78.2 ± 9.8	84.3 ± 11.6	<0.001	–4.51 (–8.06, –0.97)	0.013		–4.60 (–8.16, –1.04)	0.011

Hill Bone Compliance to High BP Therapy Scale**								

Total Hill Bone score [56]	18.4 ± 2.55	22.5 ± 8.13	<0.001	–4.57 (–7.07, –2.07)	<0.001		–4.48 (–6.97, –1.99)	<0.001

– Medication compliance score [36]	10.1 ± 1.58	12.5 ± 5.76	0.001	–3.01 (–4.92, –1.10)	0.002		–2.94 (–4.84, –1.03)	0.003

– Salt related score [12]	5.47 ± 1.36	6.59 ± 1.56	<0.001	–1.18 (–1.75, –0.62)	<0.001		–1.18 (–1.75, –0.62)	<0.001

– Appointment related score [8]	2.91 ± 1.02	3.43 ± 1.48	0.013	–0.38 (–0.82, 0.058)	0.95		–0.38 (–0.82, 0.66)	0.091

Medication adherence self-efficacy score [52] ***	50.6 ± 2.01	46.7 ± 9.74	0.001	3.94 (0.55, 7.33)	0.023		3.86 (0.49, 7.23)	0.025

Knowledge of hypertension	20.7 ± 2.39	18.5 ± 4.68	<0.001	1.81 (0.39, 3.24)	0.013		1.73 (0.32, 3.15)	0.016


*Note*: M (SD) = Mean (Standard Deviation); ^a^ = t-test; CI = Confidence interval. ^b^ = Results are presented as mean differences with the 95% CI at follow up (12 weeks) calculated using Mixed effect models with baseline value and control group as reference categories. Model 1: Unadjusted; Model 2: adjusted for age, sex, education, marital status, occupation, religion, smoking, alcohol intake. ** the lower the score, the higher the adherence *** the higher the score, the better the medication adherence self-efficacy.

**Figure 3:**
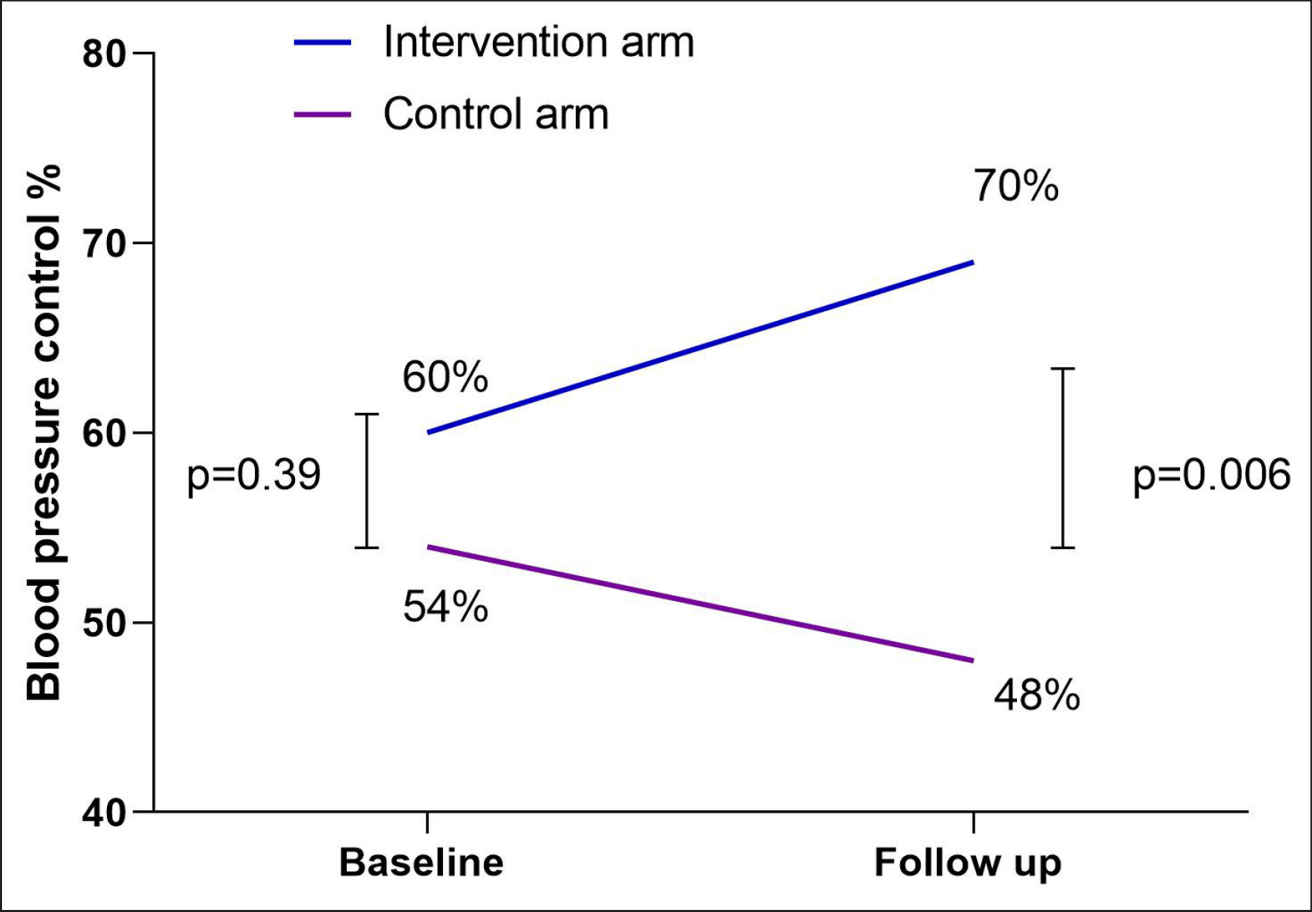
Blood pressure control at baseline and follow-up in the text message intervention and control groups.

BP control (<140/90 mm of Hg) improved by 10% in the intervention arm, but this was not observed in the control arm (–6.00%) with a substantial difference of 70% vs 48% control (p = 0.006) at follow-up (***Figure 3***)

#### Hill Bone compliance score

**Figure 4:**
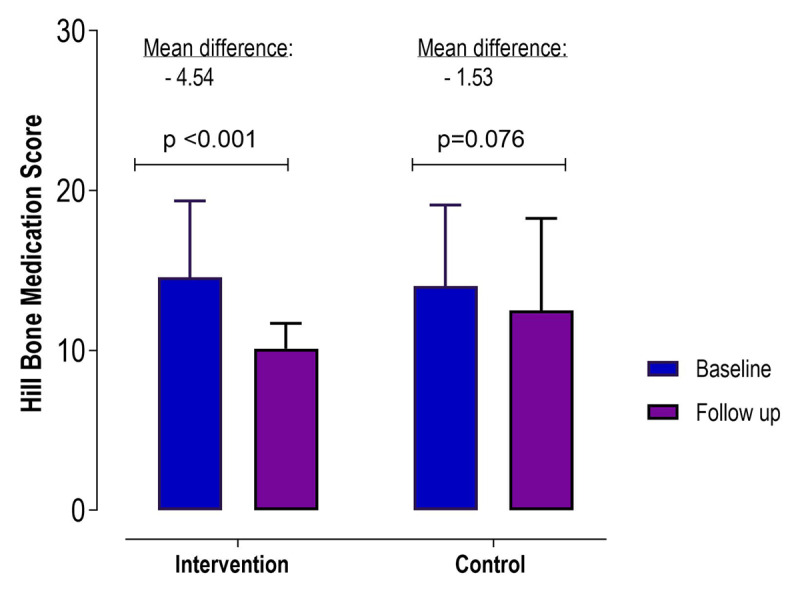
Change in Hill Bone medication compliance-related score from baseline to follow-up in the text message intervention group compared to the control group.

In-group analysis showed a decrease in the Hill Bone medication compliance score only in the intervention arm (p < 0.001) at follow up, with a lower score reflecting increased medication adherence (***Figure 4***).

At follow-up, we found a significantly lower total Hill Bone score (p < 0.001) in the intervention arm compared to the control arm. The total Hill Bone compliance score (β = –4.57, p < 0.001), medication-related score (β = –3.01, p < 0.001), and salt-related score (β = –1.18, p < 0.001) showed greater reductions among the intervention arm at follow-up compared to the control arm and remained significant also after adjustment of other covariates. However, there was no change in the Hill Bone appointment-related score (***Table 2***).

#### Medication adherence self-efficacy

The score improved more among the intervention arm (β = 3.94, p = 0.023) than the control arm at follow-up before and after adjustment for covariates (***Table 2***).

#### Knowledge Score

The total knowledge of hypertension score (β = 1.81, p = 0.013) improved slightly among the intervention arm at follow-up compared to the control arm, also after adjustment (***Table 2***).

In addition, correlation analysis of the primary and secondary outcomes showed that, overall, these outcomes were correlated. It showed that DBP showed borderline correlations with the Hill Bone medication related score (r = 0.16; p = 0.051) and negatively correlated with knowledge (r = –0.14, p = 0.075) and medication adherence self-efficacy (r = –0.12; p = 0.139). However, the medication adherence score was strongly and significantly negatively correlated with the medication adherence self-efficacy score and knowledge of the disease and treatment as a lower medication adherence score indicates higher adherence (Supplementary file 2: Stable2).

#### Sensitivity Analyses

We also used multiple imputations to impute missing values at follow-up and performed general linear univariate and multivariate analyses using the imputed data (Supplementary File 2: STable 3). These analyses corroborate the findings in ***Table 2*** regarding the effectiveness of the intervention.

### Acceptability of the intervention

At follow-up, most of the participants in the intervention arm responded that they found the text messaging useful (89%), culturally appropriate (90%), and age-appropriate (90%), and would recommend text messages to other patients with hypertension (89%) (***Table 3***). The acceptability of the text messages was not different based on literacy, sex or occupation. However, elderly participants responded that they did not find messages useful and would not recommend messages to others compared to other age groups, which was significant (Stable 4). In addition, most responded that the intervention had a positive impact on their BP (86%), impacted on eating a low salt, low-fat diet (87%), positively impacted knowledge on hypertension (89%) and had a positive impact on their physical activity (73%). However, three-quarter (74%) responded that the intervention had not impacted the frequency of monitoring their BP (***Table 3***).

**Table 3:** Responses on the acceptability of the TEXT4BP intervention (n = 79).


DO YOU THINK THOSE TEXT MESSAGES WERE	YES N (%)	NO N (%)

**Useful, n**	70 (89)	9 (11)

**Culturally appropriate, n**	71 (90)	8 (10)

**Age appropriate, n**	71 (90)	8 (10)

**Would you recommend messages to others, n**	70 (89)	9 (11)

**Do you think those text message has had a positive impact on your**		

**Overall BP, n**	68 (86)	11 (14)

**Diet or eating low salt and fat diet, n**	69 (87)	10 (13)

**Physical activity patterns, n**	58 (73)	21 (27)

**Knowledge of hypertension, n**	70 (89)	9 (11)

**Frequency of monitoring BP, n**	21 (22)	58 (78)


We used a Marshfield questionnaire to assess the intervention’s usability among the study participants. More than half (58%) of the participants strongly agreed that the SMS was easy to use (mean 4.46) and were confident in reading the message (mean 4.31). Similarly, 65% of participants strongly agreed that they could trust the message content (mean 4.63), and 61% wanted to receive the message again. Almost all responses had a mean score of more than four, where the highest mean was 4.63, indicating they were satisfied with the SMS system. The lowest mean score was 3.96, indicating that it was not as satisfying as talking to a real person (Supplementary File 2: STable 5).

The findings of the qualitative interviews were aligned with the Marshfield questionnaire. Participants found the intervention acceptable, informative and useful in supporting their behaviour modification. Participants found the format, frequency and content of the messages very acceptable. Some of the participant feedback included: *‘It is simple…It’s in understandable form’ (P01:18–30yr, male, literate)*, and *‘I really felt good to receive text messages because I got to know about unknown things like—we should not take this food; we should carry medicine while travelling etc. I feel like I am receiving necessary information. It is good’ (P03:31–45yr, female, literate)*.

Participants also expressed that message related to preferred food during festival times were very helpful in controlling the high salt and high-fat foods. Some participants shared that they felt that someone was caring about their health when they received the messages. Some participants also expressed that text messages reminded them to take and buy medicine on time: *‘it worked as a reminder to me’ (P 04:46–60yr, male, illiterate)*. Some also expressed their desire to continue the program. However, illiterate participants expressed difficulties in reading the messages. The family member was supposed to read the text message for them, but this was deemed to be problematic in some cases due to the unavailability of the family member, e.g., *‘The problem is; my son was occupied with work. Due to his job, he wasn’t able to meet me and read message for me’ (P05:60–69yrs, female, Illiterate)*. Participants suggested the use of voice messaging to overcome the challenges of illiteracy.

## Discussion

This is the first randomised controlled trial to our knowledge that used the text messaging mHealth for the management of hypertension in Nepal. Our study found a culturally acceptable mobile phone text messaging intervention to be effective in reducing systolic and diastolic BP and improving BP control when compared to usual care in an LMIC, Nepal. The study demonstrated improved adherence to antihypertensive medication, including medication adherence self-efficacy, and a modest improvement in knowledge regarding hypertension among participants receiving text messages. We found that the intervention was highly acceptable to participants, and participants expressed a desire for the continuation of the program. Collectively, our study demonstrates that mHealth can be a viable and practical approach in LMICs and has the potential to be integrated into the wider health system. This is in line with the Nepal Health Sector Strategy 2015–2020 [36] which stressed the importance of evidence-based use of modern technology in health services and information delivery in Nepal. Our findings should be confirmed in a large, randomised trial across multiple settings with long-term follow-up to demonstrate cost-effectiveness.

In LMICs, the use of simple text messages can contribute to bridging the gap in health care access especially in populations of ethnic minorities [37,38]. Our findings are encouraging and corroborates a systematic review and meta-analysis (Text2preventCVD) that showed a greater improvement of SBP –4.13 mm Hg, and DBP –1.11 mm Hg in studies using text messages [39]. However, all included trials did not focus on patients with hypertension. Another recent systematic review and meta-analysis that focused on 24 randomised trials using mHealth interventions among hypertensive patients provided evidence of a greater reduction of both SBP –3.78 mmHg and DBP –1.57 mmHg among the mHealth intervention groups [40]. However, most of these studies (22) were from HICs, and only 10 studies used text messages as an intervention; the other studies used interventions based on apps and other types of mHealth. The STAR Trial conducted in South Africa [41] among patients with hypertension reported a small change in SBP (–2.2 mm Hg) at 12 months compared to our study (–6.36 mmHg) assessed at 3 months. Although we were not able to report on the long-term effectiveness of the intervention in our study, a previous systematic review reported no difference on the effect of the text messaging interventions based on duration [42].

The mechanism of effectiveness in improvement in BP might be through greater patient engagement in self-care through text messages by continuation of care beyond the hospital setting [43]. This mechanism may have filled the continuity-in-care gap between health workers and patients in low resource settings by providing acceptable information to adopt a healthy lifestyle [44]. Additionally, messages informed by the theoretical model have the advantage of catering for the needs of and ensuring better engagement with patients [45] rather than only transferring knowledge [46]. Our theory-informed intervention resulted in better patient engagement and motivation for behaviour change leading to effectiveness of the intervention in our study.

Medication non-adherence is one of the major predictors of uncontrolled BP among patients with hypertension [47]. Text messages act as reminders to reinforce daily taking of medication [17] and address the issue of forgetfulness, which is identified as one of the barriers for medication adherence [48]. Additionally, higher self-efficacy is associated with better initiation and engagement with self-care behaviours such as medication adherence, physical activity and dietary changes among patients with hypertension [49,50]. Similar improvements of medication adherence were reported in previous systematic reviews of patients with chronic disease [42] and among patients with hypertension [51] using text message interventions. However, most of the included studies were from high-income countries. It is well established that the level of education and employment, dietary habits and preferences, lifestyle behaviours and a general understanding on the risks of raised BP [23] are substantially different in LMICs, and thus findings from high income countries cannot be directly translated to low resource settings.

There is very little evidence on the acceptability of text message interventions in the self-management of long-term illness [51]. Our study addressed this gap by providing evidence that a contextual co-designed mHealth intervention can be acceptable, useful and informative in low resource settings. Participants acknowledged their interest in continuation of the program beyond the follow-up time. In the Text2PreventCVD systematic review most studies reported useful and moderate to high levels of satisfaction with a text-messaging programme [39]. One study reported that young participants may be more ready to accept text messaging interventions [52], where others reported a decline in the interest of the participants over time [53]. Also in our study, there was a hesitancy in acceptance of the intervention among illiterate participants, who found it difficult to read the text messages. Further studies are recommended to determine the long-term acceptability of our mHealth intervention.

Our study provides clear evidence on the effectiveness and acceptability of text messaging interventions in patients with hypertension in Nepal, despite the study coinciding with a festival season in Nepal (Dashain and Tihar) where high fat, high salt diets and excessive alcohol use are considered celebrated foods. The effectiveness of the messages in our study may be due to the impact of theory driven co-designed contextual text messages (including the impact of festive food and local herbs) through formative studies, which was acknowledged by the study participants. Furthermore, in times of COVID-19, integration of mHealth interventions for the management of chronic illness in healthcare settings has become more relevant than ever.

Data security and ethical aspects need special consideration during implementation of mHealth or other technology-based interventions. The potential harm of such interventions includes the likelihood of exposing personal information, less accountability of the collected information from the concerned stakeholder due to the restricted role, and capacity of the ethics committee at LMICs [54]. However, there is limited evidence of any harms in LMIC settings, which is an area of further research.

### Strengths and limitations

Our intervention development followed a vigorous co-designed method, informed by the COM-B behaviour change model [29] and evidence of formative studies [22,23], which ensured the robustness of our intervention. We have previously described the details of the intervention and delivery [24] that can be replicated. By using randomisation, we reduced selection and allocation bias. There are also limitations that need to be considered. Our pilot study was not powered to detect the observed difference of BP between groups though our sample size decision was based on the standard recommendation for the pilot RCT [25]. We used self-reported measures for assessing medication adherence which may have overestimated adherence. However, we used the validated Hill Bone tool with high reliability to overcome this limitation. This trial was not blinded, but we minimised potential bias by conducting group-allocation after the completion of baseline data collection. We minimised measurement bias at follow-up by advising the study participants not to disclose their allocation until measuring their outcomes at follow up. This was a small-scale intervention conducted at a tertiary referral-level hospital, thus generalisability of our findings to remote areas is unclear.

## Conclusion

Our TEXT4BP randomised controlled trial has demonstrated that a contextual co-designed text messaging intervention informed by the behaviour change model to reduce BP and improve control in low resource settings is feasible, acceptable and effective. Large-scale controlled trials building on our findings are recommended to evaluate the implementation of text message interventions in broader communities in LMICs to determine the sustainability of the effectiveness and acceptability.

## Data Accessibility Statement

Data associated with this paper is available upon submitted request to the corresponding author.

## Additional Files

The additional files for this article can be found as follows:

10.5334/gh.1103.s1 Supplementary Files 1. Example of development of TEXT4BP text messages based on the formative qualitative study findings using Behaviour change techniques.

10.5334/gh.1103.s2 Supplementary Files 2. Stables 1–5.
