# Supplementary material for: Effectiveness and Acceptability of a Mobile Phone Text Messaging Intervention to Improve Blood Pressure Control (TEXT4BP) among Patients with Hypertension in Nepal: A Feasibility Randomised Controlled Trial
Source: Glob Heart. 2022 Feb 23;17(1):13. doi: 10.5334/gh.1103 (PMC8877709; doi:10.5334/gh.1103)
Supplement: Supplementary Files 1. — Example of development of TEXT4BP text messages based on the formative qualitative study findings using Behaviour change techniques. [file gh-17-1-1103-s1.pdf]

Example of development of TEXT4BP text messages based on the formative qualitative study findings using Behaviour change techniques

**Barriers /facilitators**	**Linking with BCTs**	**Content of text message**
**Capability barriers**<br>Literacy of hypertension and its treatment	Shaping knowledge<br><br>Health consequences	Do you know? You may have no symptoms and still have high blood pressure. Remember to take your medicine regularly.<br><br>Do you know? Uncontrolled Blood pressure which is above 140/90 puts you in danger of having complications. Remember to check your BP regularly.
**Motivation barriers/facilitators**<br>Beliefs about consequences of diseases<br>Faith in traditional medicine/ local herbs	Threat<br>Negative reinforcement<br><br>Shaping knowledge	Uncontrolled High blood pressure can lead to heart attack, paralysis, vision problems and kidney failure so take your medicine regularly.<br><br>Do not only rely on local remedies like bitter things to control your blood pressure. There are no other supplements to blood pressure medication. You must take medicine if your doctor prescribed it.
**Capability/Opportunity/motivation/ barriers**<br>Non-adherence<br><br>Forgetting to take medicine	Reinforcement<br>Shaping knowledge/ Emotional consequences<br><br>Prompts /Cues<br><br>Habit formation	Are you taking your medicine regularly? Remember! NEVER change your medication or stop taking your medication unless your doctor tells you to.<br>If you are not sure you really need your blood pressure medicine, ask your doctor to explain the reasons why it was prescribed.<br>Try putting your pillbox or bottles near something you see every day, like your toothbrush to help remember to take your blood pressure medication!<br>Do you know? you can set up an alarm on your mobile phone to remind you to take medications
**Opportunity/motivation barriers**<br><br>**unhealthy dietary habits (cultural practices)**	Shaping knowledge<br><br>Self-monitoring of behaviour	Did you know? Smoking, drinking alcohol, eating unhealthy food (high salt and high fat food), little exercise and being mentally stressed puts you in danger of high blood pressure.<br>Did you exercise this week? Aim at exercising 30 minutes/day at least five times a week.<br><br>**Salt intake:** Foods high in sodium(salt) can increase your blood pressure. Try to limit your sodium intake to 5g/day, including what is in and what is added to food.

**Resistance in behaviour modification/**	Goal setting	**Festive food:** Happy Dashain/Happy Tihar *(Nepali festival name which was during intervention).* Please remember to enjoy the festival limiting food with high salt and high fat which will affect your blood pressure.
	Shaping knowledge	**Physical activity:** Moderate physical activity of 30 minutes can make your blood pressure medications work more efficiently and reduce your blood pressure. Try a 30-minute brisk walk or three 10-minute walks
**Stigma and Non disclosure**		Don't feel ashamed in disclosing your high blood pressure status. Please disclosure yours and encourage other to disclose for the timely diagnosis and seek treatment to prevent the complications.
	Reinforcement	Don't worry! If you have high blood pressure, it does not mean you are ill or weak. If you can keep the blood pressure under control (<140/90) then it will not harm you.
