# Supplementary material for: Effectiveness and Acceptability of a Mobile Phone Text Messaging Intervention to Improve Blood Pressure Control (TEXT4BP) among Patients with Hypertension in Nepal: A Feasibility Randomised Controlled Trial
Source: Glob Heart. 2022 Feb 23;17(1):13. doi: 10.5334/gh.1103 (PMC8877709; doi:10.5334/gh.1103)
Supplement: Supplementary Files 2. — Stables 1–5. [file gh-17-1-1103-s2.pdf]

Supplementary tables

STable 1: Comparison of demographic profile of completers and loss to follow-up group

Characteristics	Category	Completers N =154 (%)	Los to follow up N= 46 (%)	p value
**Age**	Mean ± SD	50.2 ± 9.79	51.3 ± 8.80	0.35
**Sex**	Male	84(54.5)	27(58.7)	0.63
**Literacy status**	Literate	118(76.6)	36(78.3)	0.82
**Ethnicity**	Upper caste groups	91(59.1)	32(69.6)	0.36
	Advantages/disadvantages Janajatis	54(35.1)	11(23.9)	
	Others	9 (6.5)	3 (5.8)	
**Marital status**	Currently married	136(88.3)	44(95.7)	0.15
**Occupation**	Employed	83(53.9)	25(54.3)	0.96
**Religion**	Hindu	139(90.3)	44 (95.7)	0.25
	Others	15(9.7)	2(4.3)	

STable 2: Correlation of the primary and secondary outcomes of the study at follow up

Variables	Systolic BP	Diastolic BP	Hill Bone Medication adherence score	Medication adherence self-efficacy score	Knowledge of hypertension
Systolic BP	1	r= 0.69<br>p=<0.001	r= 0.02<br>p=0.830	r= - 0.02<br>p=0.806	r= - 0.11<br>p=0.159
Diastolic BP	r=0.69<br>p=<0.001	1	r= 0.16<br>p=0.051	r= - 0.12<br>p=0.139	r= - 0.14<br>p=0.075
Hill Bone Medication adherence	r= 0.02<br>p=0.83	r= 0.16<br>p=0.051	1	= - 0.78<br>p==<0.001	= - 0.56<br>p==<0.001
Medication adherence self-efficacy score	r= - 0.02<br>p=0.806	r= - 0.12<br>p=0.139	= - 0.78<br>p==<0.001	1	= 0.55<br>p==<0.001
Knowledge of hypertension	r= - 0.11<br>p=0.159	r= - 0.14<br>p=0.075	=- 0.56<br>p==<0.001	= 0.55<br>p==<0.001	1

STable 3: Effectiveness of TEXT4BP intervention among the intervention arm compared to control arm using data based on multiple imputation

Outcome variable	Regression coefficient* Model 1		Regression coefficient* Model 2	
	**β (95% CI)**	***p value***	**β (95% CI)**	***p value***
**Systolic blood pressure**	-8.56 (-12. 97, -4.14)	<0.001	-8.4 (-12.9, - 3.9)	<0.001
**Diastolic blood pressure**	- 5.40 (-8.35, -2.45)	<0.001	-5.28 (-8.33, -2.23)	0.001
**Hill Bone compliance scale** **Total score**	-4 (-6.15, -1.84)	<0.001	-4.29 (-6.48, -2.11)	<0.001
**- Medication related score**	-2.40 (- 3.89, -0.91)	0.002	-2.56 (- 4.07, -1.05)	0.001
**- Salt related score**	-1.12 (-1.63, -0.58)	<0.001	-1.05 (-1.59, -0.51)	<0.001
**- Appointment related score**	-0.45 (-0.88, 0.03)	0.37	-0.50 (-0.94, -0.06)	0.024
**Medication adherence self-efficacy**	3.01 (1.23, 4.79)	0.001	3.20 (1.39, 5.01)	0.001
**Knowledge of hypertension**	1.58 (0.62, 2.54)	0.001	1.50 (0.53, 2.46)	0.002

***** General linear univariate and multivariate model of imputed data Model 1: Unadjusted model,

Model 2: Adjusted model for age, sex, education, marital status, occupation, religion, smoking status, alcohol intake, BMI.

STable 4: Association of demographic factors with the acceptability of the TEXT4BP intervention (n=79)

**Characteristics**	**Category**	**Useful**			**Culturally appropriate**			**Age appropriate**			**Recommend messages to others**		
Age		Yes 70 (89)	No 9 (11)	p[a]	Yes 71 (90)	No 8 (10)	p[a]	Yes 71 (90)	No 8 (10)	p[a]	Yes 70 (89)	No 9 (11)	p[a]
	18 – 45	24 (94)	2 (6)	0.024*	24 (94)	2 (6)	0.206	24 (94)	2 (6)	0.206	24 (94)	2 (3)	0.024*
	46 – 60	40 (93)	3 (7)		40 (93)	3 (7)		40(93)	3 (7)		40(93)	3 (7)	
	60 -69	6(60)	4 (40)		7(70)	3(30)		7(70)	3(30)		6(60)	4 (40)	
Sex	Male	42 (89)	5 (11)	0.798	43 (91)	4 (9)	0.807	43 (91)	4 (9)	0.807	42 (89)	5 (11)	0.798
	Female	28 (88)	4 (13)		28 (88)	4(13)		28 (88)	4 (13)		28 (88)	4 (13)	
Literacy	Literate	59(91)	6(9)	0.193	57(88)	6(9)	0.695	57(88)	6(9)	0.695	59(91)	6(9)	0.193
	Illiterate	11(79)	3(7)		12(86)	2(14)		12(86)	2(14)		11(79)	3(21)	
Occupation	Employed	38(93)	3(16)	0.236	39(95)	2(5)	0.273	39(95)	2(5)	0.273	38(93)	3(16)	0.236
	Unemployed	32(84)	6(16)		32(84)	6(16)		32(84)	6(16)		32(84)	6(16)	

[a]p value of Chi-square test, * significant $p<0.05$

STable 5: Responses on the Marshfield usability of the intervention questionnaire

S. N	Questions	Strongly agree	Agree	Neither agree nor disagree	Disagree	Strongly disagree	Mean ± SD
**1.**	I thought SMS system was easy to use	58 (73.4)	9 (11.4)	7 (8.9)	1 (1.3)	4 (5.1)	4.46±1.06
**2.**	I felt very confident reading SMS	58 (73.4)	5 (6.3)	7 (8.9)	1 (1.3)	8 (10.1)	4.31±1.30
**3.**	I needed to learn a lot of things before I could get going with SMS*	6 (7.6)	5 (6.3)	9 (11.4)	2 (2.5)	57 (72.2)	4.25±1.31
**4.**	I felt that I needed someone's help to be able to read the SMS*	8 (10.1)	7 (8.9)	7 (8.9)	4 (5.1)	53 (67.1)	4.10±1.42
**5.**	I found SMS to be complex*	5 (6.3)	3 (3.8)	8 (10.1)	2 (2.5)	61 (77.2)	4.40±1.20
**6.**	Using the system of SMS did not take much time	61 (77.2)	5 (6.3)	7 (8.9)	2 (2.5)	4 (5.1)	4.48±1.09
**7.**	I could always trust the SMS to work	65 (82.3)	3 (3.8)	8 (10.1)	2 (2.5)	1 (1.3)	**4.63±0.86**
**8.**	My privacy was protected when I used the SMS system	59 (74.7)	3 (3.8)	8 (10.1)	1 (1.3)	8 (10.1)	4.31±1.31
**9.**	Using SMS service was as satisfying as talking to a real person	42 (53.2)	12(15.2)	10 (12.7)	10 (12.7)	5 (6.3)	**3.96±1.32**
**10.**	In general, I was satisfied with the system of SMS	59 (74.7)	12(15.2)	7 (8.9)	0	1 (1.3)	4.62±0.75
**11.**	I think I would like to receive such SMS again	61 (77.2)	4 (5.1)	13(16.5)	1 (1.3)	0	4.58±0.81
**12.**	The system of SMS could help me better manage my health and medical needs	54 (68.4)	13(16.5)	8 (10.1)	4 (5.1)	0	4.48±0.87
**13.**	I could be more involved in my care by using the SMS service system	56 (70.9)	11(13.9)	9(11.4)	2 (2.5)	1 (1.3)	4.50±0.88

**Reverse coding*
